# Hypoglycemia compensation mechanisms in dry fasting

**DOI:** 10.1016/j.metop.2025.100363

**Published:** 2025-04-15

**Authors:** Ioannis-Eleemon Papagiannopoulos-Vatopaidinos, Maria I. Papagiannopoulou, Eleni N. Dotsika

**Affiliations:** a Vatopaidi Monastery Hospital, Mount Athos, 63086, Greece; b Medical Office for Fasting Therapy, Danaidon 24a, Halandri, 15232, Greece; c Cellular Immunology, Hellenic Pasteur Institute, Vas. Sofias Avenue 27, 11521, Greece

**Keywords:** Insulin resistance decrease, Blood - Cell interphase, GFR increase, IGF-1 increase in dry fasting, Late leptin increase in dry fasting, Selective LDL increase in dry fasting, Tissue protection

## Abstract

**Background:**

Dry fasting (DF) presents three primary risks: hypovolemia, hypertonicity, and hypoglycemia. The first two have been shown to be effectively compensated, and the respective mechanisms have been studied. The behavior of glucose has only been roughly described, while the hypoglycemia compensation mechanisms remain unexplored.

**Objectives:**

Studying the glucose behavior, the hypoglycemia compensation mechanisms, and the insulin resistance during DF.

**Methods:**

Following parameters were daily monitored in ten participants undergoing a 5-day DF: Weight, body circumferences, glucose, creatinine clearance (GFR), insulin, HOMA-IR, acetoacetate in 24-h urine, glucagon, growth hormone (GH), IGF-1, TSH, T_4_, T_3_, leptin, cholesterol, LDL-cholesterol (LDL-C), HDL-cholesterol (HDL-C), triglycerides, and the enzymes LDH, CPK, SGPT, SGOT, and γGT.

**Results:**

Weight, body circumferences, TSH, T_3_, and T_4_ decreased to minima on Day 5; insulin and HOMA-IR decreased, reaching minima on Day 4; GH, cholesterol, LDL-C, and acetoacetate increased to maxima on Day 5; Glucagon, IGF-1, and GFR increased, presenting maxima on Day 4; Glucose, leptin, and triglycerides exhibited biphasic profiles with minima on Days 3, 3, and 2, respectively; HDL-C, LDH, CPK, SGPT, SGOT, and γGT showed minimal or non-significant changes.

**Conclusion:**

A comprehensive description of glucose behavior and the hypoglycemia compensation mechanisms in DF were presented. DF decreased insulin resistance, likely by improving the blood – cell interphase, and enhanced GFR. The increase in LDL-C, tissue-protecting IGF-1, and late increase in leptin and triglycerides were unexpected. The results may inform the development of novel therapeutic approaches for obesity, metabolic syndrome, type-2-diabetes, non-alcoholic fatty liver disease, adiposity, and atheromatous diseases.

## 1 Introduction

Dry fasting (DF), the most intensive form of fasting described so far [1], is defined as the voluntary abstinence from any food or fluid for a certain period of time [2]. Despite its historical and cultural significance, the physiological mechanisms underlying DF homeostasis remain insufficiently explored. Recent studies have highlighted its numerous metabolic and therapeutic implications. However, crucial aspects—particularly glucose dynamic and its endocrine context—remain largely unexplored.

The present study aims to address this gap by providing a comprehensive description of glucose behavior during DF and a detailed analysis of its hormonal and biochemical background.

### 1.1 Historical applications of DF

The practice of DF can be traced back to the 5th century B.C., when Hippocrates employed it for the treatment of acute maladies and paroxysmal episodes of chronic diseases [3]. During the Byzantine era, prominent ascetics applied DF as a valuable practice for both physical and spiritual purification [4,5]. DF has been an integral part of the fasting traditions of the Christian Orthodox Church since its inception and continues to be widely practiced throughout the centuries to this day. In particular, at the beginning of Lent, millions of believers engage in the so-called “3-day fast”, i.e., DF for 60–72 h [6].

### 1.2 Modern clinical applications and research on DF

Over the past three decades, DF has been implemented in medical settings in Greece for the treatment of various diseases. The Greek population's familiarity with DF is largely due to its religious traditions, which incorporate DF as a well-established practice.

In the past two decades, several studies involving Greek volunteers have provided insights into DF physiology and suggested its potential therapeutic applications. A study conducted on Mount Athos identified numerous volatile compounds, including acetone, in the exhaled air of monks following the “3-day fast,” indicating metabolic shifts [6].

Another study demonstrated that engaging in DF for five consecutive days was safe and led to improvements in renal function, as well as reductions in body weight, waist circumference (WC), neck circumference (NC), chest circumference at axilla (CCA), chest circumference at nipples (CCN), hip circumference (HC), and oblique hip circumference (OHC). However, this study only provided a preliminary description of glucose behavior during DF without exploring its endocrine background. It also highlighted three potential risks of DF—hypovolemia, hypertonicity, and hypoglycemia—without analyzing the compensatory mechanisms involved [7].

A more recent study focused on the hypertonicity and hypovolemia compensation mechanisms. It provided new insights into DF-induced metabolic changes, including an indirectly demonstrated increase in cellular oxygen supply, which is associated with reduced intracellular oxidative stress. Additionally, it demonstrated the short-term antioxidant, anti-edematous, anti-inflammatory, and immune-stimulating effects of DF, suggesting promising therapeutic applications [1].

### 1.3 Objectives of the current study

A deeper understanding of DF physiology is of paramount importance, as it underlies its historical, modern, and potential future therapeutic applications. As outlined in the previous sections, however, the mechanisms compensating for hypoglycemia during DF have not yet been elucidated.

This study aims to.

• Provide a comprehensive description of glucose behavior during DF, along with its neuroendocrine, endocrine, and biochemical background.
• Investigate the complex hypoglycemia compensation mechanisms.
• Examine metabolic adaptations during DF, including the alteration of insulin resistance.
• Discuss potential target diseases and DF-based therapeutic approaches.

## 2 Design, subjects, and methods

### 2.1 Design

The present measurements were performed on blood and urine samples collected between May 11 and 16, 2014. These samples were also used to determine parameters of a previous study on DF [1]. The dietary protocol is summarized below (Table 1).

**Table 1:** The dietary protocol.

Day-interval definition: 20:00 on the previous day to 20:00 on the current day.		
DAY	Diet	Anthropometric and Laboratory Measurements
0	Habitual nutrition	Weight, BMI, NC, CCA, CCN, WC, HC, OHC, serum glucose, creatinine clearance, insulin, HOMA-IR, glucagon, acetoacetate in 24-h urine, GH, IGF-1, TSH, T4, T3, leptin, cholesterol, LDL, triglycerides, HDL, LDH, CPK, SGPT, γGT, SGOT.
1	**DF (dry fasting)**	Same as in Day 0
2	**DF**	Same as in Day 0
3	**DF**	Same as in Day 0
4	**DF**	Same as in Day 0
5	**DF**	Same as in Day 0
6	**20:00**: 300 ml juice (drunk with a tsp)	No measurements
	**07:00:** 300 ml juice (drunk with a tsp)	
	**12:00:** 500 gr. fruits (chewed 20 times per bite)	
	**16:00:** 300 ml juice (drunk with a tsp)	
7	**20:00**: 300 ml juice (drunk with a tsp)	No measurements
	**07:00:** 300 ml juice (drunk with a tsp)	
	**12:00:** 400 gr. boiled potatoes or rice along with 2–3 Tbsp olive oil and 2–4 Tbsp fresh lemon juice (chewed 20 times per bite). **Up to 300 ml of water a day**	
8	**20.00**: 250 ml juice (drunk with a tsp.)	No Measurements
	**07:00:** 400 gr. fruits (chewed 20 times per bite)	
	**12:00:** a light meal, e.g., 500 g boiled or baked food (no fried or sauteed) or fresh salat along with 3–4 Tbsp cold-pressed olive oil and 2–4 Tbsp fresh lemon juice (chewed at least 20 times per bite). **Water as less as possible.**	

At the end of Days 0–5, following parameters were measured: Weight and body circumferences; glucose, insulin, GH, IGF-1, TSH, T_4_, T_3_, leptin, cholesterol, LDL-C, HDL-C, triglycerides, and the enzymes LDH, CPK, SGOT, SGPT, and γGT in serum; glucagon in plasma; acetoacetate in 24-h urine; and creatinine clearance based on creatinine in serum and 24-h urine. HOMA-IR was calculated from insulin and glucose levels.

### 2.2 Recruitment procedure and inclusion/exclusion criteria

Ten participants (three men and seven women), who were not receiving any medication, had normal glucose metabolism, no history of diabetes mellitus, and no cardiovascular issues, were recruited from patients of a medical office specializing in fasting therapy in Athens. Recruitment was conducted in cooperation with the Hellenic Pasteur Institute. Their average age, weight, height, and BMI were 49.5 years (range 30–65), 85.3 kg (range 58–102), 1.7 m (range 1.60–1.89), and 29.5 kg/m^2^ (range 20–39), respectively.

The participants were informed about the purpose of the daily measurements and specimen collection and provided written consent before data collection. The inclusion and exclusion criteria are presented below (Table 2).

**Table 2:** Inclusion-exclusion criteria.

Inclusion-Criteria	Exclusion-Criteria
Adults between 18 and 75 years	Pituitary insufficiency
At least one experience with multiple-day dry fasting	Renal insufficiency
Being healthy and able to walk and work	Adrenal insufficiency
Receiving no medication	BMI under 19.5 kg/m^2^
Having a good communication ability	Major psychiatric and cognitive disorders such as psychoses, severe depression, mental retardation, dementia, and autism
	Type-1-diabetes mellitus and diabetes insipidus
	Active malignant diseases

#### 2.2.1 Hormonal and biochemical measurements

The immunoassay analyzer Immulite 1000 (Siemens, Germany) was employed to measure insulin, GH, IGF-1, TSH, T_4_, and T_3_. An ELISA analyzer Expert 96 (ASYSHitech, Austria) was used to quantify glucagon using microplates from R&D (USA) and leptin using microplates from Bender MedSystems (Austria). Serum glucose, serum and urine creatinine, cholesterol, LDL-cholesterol (LDL-C), triglycerides, HDL-cholesterol (HDL-C), LDH, CPK, SGPT, SGOT, and γGT were measured following standard biochemical laboratory methods (ABX Pentra 400, Horiba, France). HOMA-IR in μU∗mol/L^2^ was calculated based on serum insulin and glucose concentrations [8]. Urine acetoacetate concentration was measured as a proxy for all ketone bodies (acetoacetate, β-hydroxybutyrate, and acetone) using a semi-quantitative reflectometric analysis of urine test-strips (Urine Multistix, Siemens, Germany) and its weight in 24-h urine calculated.

### 2.3 Statistical analysis

All statistical analyses were performed using SPSS software package (SPSS for Windows, Version 15.0; SPSS Inc., Chicago Il, USA). In all figures, each mean value is presented along with its standard error bar. To determine the significance of changes in all parameters, two non-parametric tests for correlated samples, the Friedman and Wilcoxon signed-rank tests, were used. The first was used to detect overall changes across Days 0–5, whereas the second assessed changes on Day 5 compared to Day 0. The p values were denoted p_f_ and p_w_ respectively. Since glucose, leptin, and triglycerides demonstrated biphasic curves, additional Wilcoxon signed rank tests were applied on day of minimum compared to Days 0 and 5. To determine the significance of the maximal change in each parameter, the Wilcoxon signed rank test was applied on Day mx (Day mx is day of maximum) compared to Day 0 to establish the p_w0-mx_. The specific results are integrated in the corresponding figures and p_w0-mx_ values in Table 3 as well. No multiple comparisons adjustments were implemented.

**Table 3:** Mean values±standard errors (standard deviations).

Parameter Units	Day 0	Day 2	Day 3	Day 5	Mx. Ch. (stdev) [Day] Significance	Normal range
M. cum. weight change kg	0.0 ± 0.0 (0.0)			−7.01 ± 0.2 (0.6)	−7.01 ± 0.2 (0.6) [5] p_w0-5_ = 0.005	
M. cum. BMI change kg/m^2^	0.0 ± 0.0 (0.0)			−2.47 ± 0.1 (0.3)	−2.47 ± 0.1 (0.3) [5] p_w0-5_ = 0.005	
M. cum. NC change cm	0.0 ± 0.0 (0.0)			−1.8 ± 0.1 (0.2)	−1.8 ± 0.1 (0.2) [5] p_w0-5_ = 0.004	
M. cum. WC change cm	0.0 ± 0.0 (0.0)			−8.6 ± 0.5 (1.6)	−8.6 ± 0.5 (1.6) [5] p_w0-5_ = 0.005	
M. cum. CCA change cm	0.0 ± 0.0 (0.0)			−4.1 ± 0.4 (1.2)	−4.1 ± 0.4 (1.2) [5] p_w0-5_ = 0.005	
M. cum. CCN change cm	0.0 ± 0.0 (0.0)			−5.7 ± 0.4 (1.3)	−5.7 ± 0.4 (1.3) [5] p_w0-5_ = 0.005.	
M. cum. HC change cm	0.0 ± 0.0 (0.0)			−5.2 ± 0.2. (0.6)	−5.2 ± 0.2 (0.6) [5] p_w0-5_ = 0.005	
M. cum. OHC change cm	0.0 ± 0.0 (0.0)			−5.3 ± 0.6 (1.8)	−5.3 ± 0.6 (1.8) [5] p_w0-5_ = 0.005	
Glucose mg/dl	86 ± 3 (11)		**56** ± 4 (12)	78 ± 2 (7)	−30 ± 5 (16) [3] p_w0-3_ = 0.005	60–100
Creatinine clear. ml/min	**151** ± 11 (34)			**207** ± 21 (65)	98.7 ± 28 (85) [4] p_w0-4_ = 0.005	60–150
Insulin μU/ml	22 ± 3 (10)			7 ± 1 (3)	−17 ± 3 (10) [4] p_w0-4_ = 0.008	6–24
HOMA-IR. μU∗mol/L^2^	4.8 ± 0.8 (2.3)			1.4 ± 0.3 (0.8)	3.8 ± 0.8 (2.5) [4] p_w0-4_ = 0.005	0.5–1.4
Ur. acetoacetate mg/24-h	**27** ± 7 (21)			**503** ± 60 (189)	477 ± 60 (190) [5] p_w0-5_ = 0.005	<20 mg/24-h
Glucagon pg/ml	51 ± 5 (16)			86 ± 3 (10)	43 ± 6 (20) [4] p_w0-4_ = 0.005	40–130
GH ng/ml	0.9 ± 0.1 (0.2)			2.8 ± 0.3 (0.9)	1.9 ± 0.3 (0.9) [5] p_w0-5_ = 0.007	0.01–3 (m), 0.03–10 (f)
IGF-1 ng/ml	115 ± 14 (45)			179 ± 19 (61)	65 ± 25 (81) [4] p_w0-4_ = 0.012	42–488
TSH mIU/l	2.7 + 0.2 (0.8)			0.7 ± 0.1 (0.3)	−2±0.2 (0.9) [5] p_w0-5_ = 0.005	0.3–5
T4 μg/dl	8.4 ± 0.3 (1.0)			6.8 ± 0.1 (0.4)	−1.6 ± 0.3 (1.1) [5] p_w0-5_ = 0.007	5.0–12.5
T3 μg/l	1.3 ± 0.0 (0.1)			1.0 ± 0.0 (0.1)	−0.3 ± 0.0 (0.1) [5] p_w0-5_ = 0.005	0.8–1.9
Leptin ng/ml	**74** ± 24 (76)		**37** ± 15 (46)	**68** ± 21 (65)	−37 ± 32 (100) [3] p_w0-3_ = 0.012	0.7–5.3 (m), 3.3–18.3 (f)
Cholesterol mg/dl	**214** ± 12 (39)			**268** ± 17 (54)	54 ± 21 (67) [5] p_w0-5_ = 0.005	<170
LDL-cholesterol mg/dl	125 ± 12 (37)			**187** ± 15 (49)	62 ± 19 (61) [5] p_w0-5_ = 0.007	<129
Triglycerides mg/dl	**182** ± 43 (136)	100 ± 6 (20)		132 ± 9 (30)	−82 ± 43 (137) [2] **p_w0-2_=0.066**	<150
HDL-Cholesterol mg/dl	55 ± 7 (21)			56 ± 6 (19)	5 ± 10 (32) [2] **p_w0-2_=0.083**	30–65 (m), 35–85 (f)
LDH U/l	284 ± 24 (77)			320 ± 25 (78)	48 ± 38 (122) [4] p_w0-4_ = 0.019	150–450
CPK U/l	86 ± 12 (38)			94 ± 19 (60)	8 ± 22 (71) [5] **p_w0-5_=0.440**	20–200
SGPT U/l	12 ± 1 (5)			15 ± 2 (6)	3 ± 2 (8) [5] p_w0-5_ = 0.024	7–35
γGT U/l	21 ± 2 (7)			24 ± 3 (9)	3.6 ± 3.6 (12) [5] **p_w0-5_=0.05**	7–40 (m), 4–25 (f)
SGOT U/l	22 ± 2 (6)			29 ± 9 (27)	7 ± 9 (28) [5] **p_w0-5_=0.86**	15–40 (m), 10–30 (f)

In all statistical analyses, a two-tailed p < 0.05 was considered significant.

## 3 Results

Symptoms and all critical clinical and laboratory findings during DF have been previously presented [1], whereas the current results are summarized below (Table 3).

### 3.1 Anthropometric parameters

Weight, BMI, WC, NC, CCA, CCN, HC, and OHC exhibited highly significant overall changes and significant decreases on Day 5 (day of minima) compared to Day 0 (Table 3).

### 3.2 Serum glucose and creatinine clearance (GFR)

**Fig. 1:**
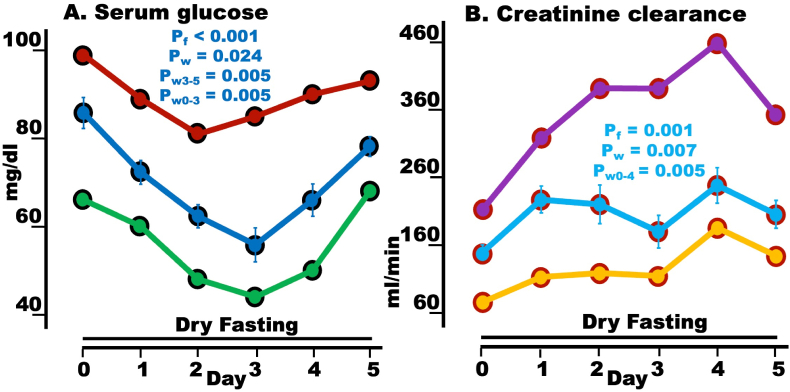
Hypoglycemia compensation and GFR (creatinine clearance) during dry fasting (DF). A glucose in mg/dl (1 mg/dl = 0.056 mmol/L); **B** creatinine clearance in ml/min. The graphs present minimum, maximum, and mean values (with standard error bars) in absolute scales, before and during DF.

Glucose demonstrated a biphasic pattern, with a highly significant overall change and a minimum on Day 3. Significant decreases were found on Days 3 and 5 compared to Day 0, whereas a significant increase was recorded on Day 5 compared to Day 3 (Fig. 1A, Table 3).

Out of fifty glucose values acquired during DF (Days 1–5), only a minority fell below 60 mg/dl. Moreover, these subnormal values were not related to any major symptoms in participants. This can be interpreted as a state of actual euglycemia.

Creatinine clearance (GFR) presented a highly significant overall change and significant increases on Days 4 and 5 compared to Day 0. The maximal increase was found on Day 4 and amounted 98.7 ± 28 ml/min in absolute and 65.4 ± 23.5 % in percent scale (Fig. 1B, Table 3).

### 3.3 Insulin and HOMA-IR

**Fig. 2:**
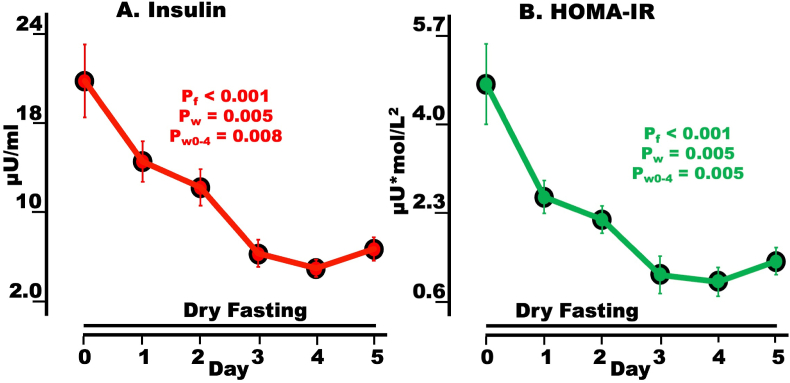
Hypoglycemia defense and insulin resistance during dry fasting (DF). A serum insulin in μU/ml; and **B** HOMA-IR (homeostasis model assessment of insulin resistance) in μU∗mol/L^2^. The graphs present mean values (with standard error bars) in absolute scales, before and during DF.

Insulin exhibited a highly significant overall change and showed significant decreases on Day 4 (day of minimum) and Day 5 compared to Day 0 (Fig. 2A**,** Table 3).

HOMA-IR exhibited a highly significant overall change and significant decreases on Day 4 (day of minimum) and Day 5 compared to Day 0 (Fig. 2B, Table 3).

### 3.4 Acetoacetate in 24-h urine, glucagon, growth hormone, and IGF-1

**Fig. 3:**
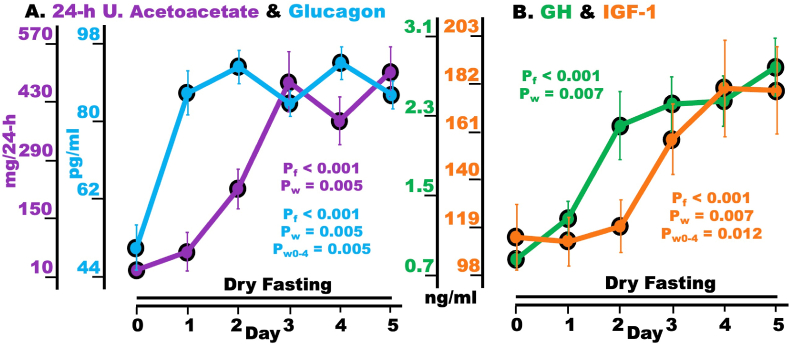
Hypoglycemia defense and tissue protection during dry fasting (DF). A acetoacetate in 24-h urine (mg/24-h) and plasma glucagon [pg/ml (pg/ml = 0.287 pmol/L)]; and **B** serum GH [ng/ml (ng/ml = 1.18 nmol/L)], and IGF-1 [ng/ml (ng/ml = 0.13 nmol/L)]; 24-h U., 24-h urine; GH, growth hormone; IGF-1, insulin-like growth factor-1.The graphs present mean values (with standard error bars) in absolute scales, before and during DF.

Acetoacetate in 24-h urine demonstrated a highly significant overall change and a significant increase on Day 5 (peak-day) compared to Day 0 (Fig. 3A, Table 3).

Glucagon exhibited a highly significant overall change and significant increases on Day 4 (peak-day) and Day 5 compared to Day 0 (Fig. 3A, Table 3). GH showed a highly significant overall change and a significant increase on Day 5 (peak-day) compared to Day 0. IGF-1 also exhibited a highly significant overall change and significant increases on Day 4 (peak-day) and Day 5 compared to Day 0 (Fig. 3B, Table 3).

### 3.5 TSH, T4, T3, and leptin

**Fig. 4:**
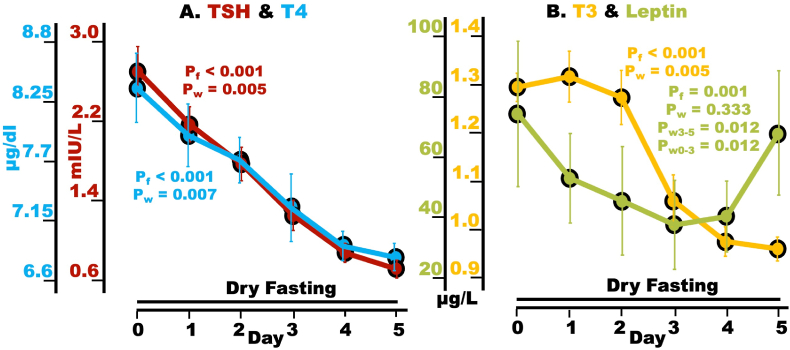
Hypoglycemia defense during dry fasting (DF). A serum TSH (mIU/l) and T_4_ [μg/dl, (μg/dl = 12.87 nmol/l)]; **B** T_3_ [μg/l, (μg/l = 1.54 nmol/l)], and leptin [ng/ml, (ng/ml = 62.5 pmol/l)]. The graphs present mean values (with standard error bars) in absolute scales, before and during DF.

TSH, T_4_, and T_3_ demonstrated highly significant overall changes and significant decreases on Day 5 (day of minima) compared to baseline (Fig. 4A and B, Table 3).

Leptin's graph exhibited biphasic pattern with a highly significant overall change, a significant decrease on Day 3 (day of minimum) compared to Day 0, and a significant increase on Day 5 compared to Day 3. A non-significant change was observed on Day 5 compared to Day 0 (Fig. 4B, Table 3).

### 3.6 Total-, LDL-, and HDL-C and triglycerides

**Fig. 5:**
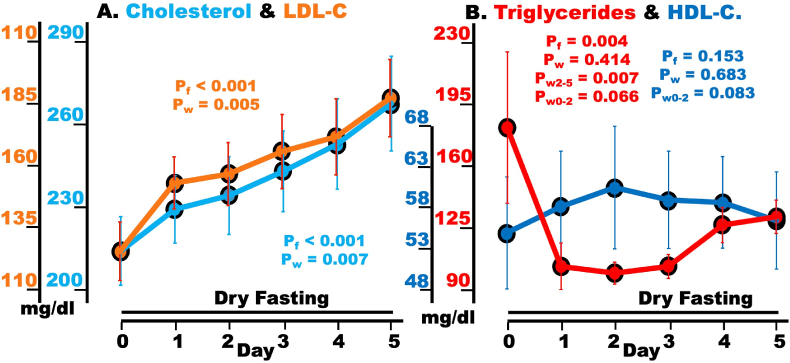
Hypoglycemia defense during dry fasting (DF). A Serum concentration of total cholesterol and LDL-C [both in mg/dl (mg/dl = 0.0259 mmol/L)] and LDL; and **B** HDL-C [mg/dl (mg/dl = 0.0259 mmol/L)] and triglycerides (mg/dl). LDL-C, low-density lipoprotein–cholesterol; HDL-C, high-density lipoprotein–cholesterol. The graphs present mean values (with standard error bars) in absolute scales, before and during DF.

Total cholesterol and LDL–C exhibited highly significant overall changes. Both showed significant increases on Day 5 (peak-day) compared to Day 0 (Fig. 5A, Table 3).

Triglycerides exhibited biphasic behavior with a minimum on Day 2, a significant overall change, and a significant increase on Day 5 compared to Day 2. Non-significant changes were recorded on Days 5 and 2 compared to Day 0. HDL–C presented no significant changes in any comparison (Fig. 5B, Table 3).

It is remarkable, that over the 5 DF-days, the increase in total cholesterol was 54 ± 21 mg/dl and in LDL-C 62 ± 19 mg/dl, while HDL-C remained unchanged (Table 3). This suggests that the body selectively increases LDL-C, leaving unchanged the HDL-C, and rather decreasing the VLDL-C.

### 3.7 LDH, CPK, SGPT, γ-GT, & SGOT

**Fig. 6:**
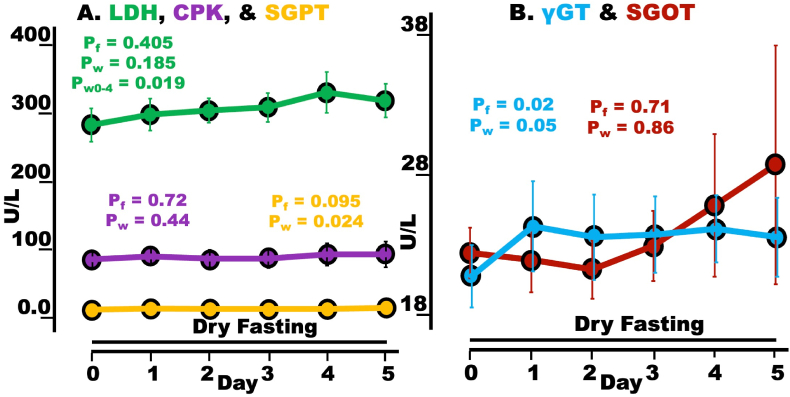
Tissue protection during dry fasting (DF). A Serum LDH, CPK, and SGPT (all in U/l); **B** Serum γGT and SGOT (both in U/l). LDH, lactate dehydrogenase; CPK, creatine phosphokinase; SGPT, glutamate pyruvate transaminase; γGT, gamma-glutamyl transferase; SGOT, glutamate oxaloacetate transaminase; The graphs present mean values (with standard error bars) in absolute scales, before and during DF. For SGPT, standard error bars were too small to be visible.

CPK and SGOT displayed non-significant changes and non-significant maxima. LDH presented non-significant changes, with a significant increase on Day 4 (peak-day) compared to Day 0. SGPT demonstrated a non-significant overall change with a significant increase on Day 5 (peak-day) compared to Day 0. Inversely, γGT showed a significant overall change with a non-significant increase on Day 5 (peak-day) compared to Day 0. Synoptically, changes in all five enzymes were either minimal or non-significant (Fig. 6A and B, Table 3).

## 4 Discussion

### 4.1 Glucose

The glucose levels shown above (Fig. 1) were impacted by (a) the discontinuation of external fueling and (b) the action of hypoglycemia compensation mechanisms. This action was present from Day 1 and included the effects of decreasing insulin, T3, and T4 and increasing hypothalamic-sympathetic efferent outflow, glucagon, GH, IGF-1, vasopressin [1], cortisol [1], noradrenaline [1], dopamine [1], ketone bodies, and LDL-C. It compromised the decrease of glucose values in Days 1–3 and effected its increase in Days 4–5. The achieved actual euglycemia (§3.2) represents a significant success of hypoglycemia compensation mechanisms, whereas the absence of any major symptoms in participants with subnormal values (§3.2) suggests an enhanced cellular tolerance to hypoglycemic values during DF.

### 4.2 GFR

The increased GFR appears to result from elevated vasopressin during DF [1]. This hormone generates a steeper axial corticomedullary osmotic gradient [9], thus upgrading both kidney's concentration ability and GFR. In combination with the slight increase in serum creatinine during DF [1,7], the increase in creatinine clearance showed a considerable increase in creatinine discharge in 24-h urine, suggesting enhanced protein catabolism. Given the minimal or non-significant changes of tissue enzymes, the origin of catabolized proteins has to be searched for.

### 4.3 Hypoglycemia sensing and neuroendocrine responses

An apparently slow-onset hypoglycemia developed during DF (Fig. 1A), which was well compensated throughout Days 1–5. Peripheral glucosensors detecting slow-onset hypoglycemia are located, among others, in mesenteric–portal vein walls [10] and pancreatic β-cells. Central glucosensors include the tanycytes and neurons expressing cholecystokinin. Tanycytes are glial cells lining the third ventricle, which relay output to neurons in the arcuate nucleus [11]. Cholecystokinin-expressing neurons are located in the lateral parabrachial nucleus in the brain stem and relay information to neurons expressing steroidogenic-factor 1 (SF1) in the hypothalamic ventromedial nucleus [12]. SF1-neurons then project to both the paraventricular nucleus (PVN) and anterior bed nucleus of the stria terminalis (aBNST). PVN responds by secreting CRH and vasopressin, both of which trigger serially the secretion of ACTH and cortisol. In turn, cortisol, particularly via intra-adrenal portal circulation, promotes the secretion of dopamine and noradrenaline. All four, vasopressin, cortisol, dopamine, and noradrenaline contribute to hypoglycemia compensation [1]. PVN and aBNST, via the rostral ventrolateral medulla and intermediolateral column, initiate sympathetic efferent outflow to pancreas, and liver (hypothalamic-sympathetic efferent outflow), resulting in inhibition of insulin secretion and increase of glucagon and hepatic glucose secretion, with decreased insulin itself stimulating glucagon secretion [[12], [13], [14]].

### 4.4 Role of insulin, glucagon, GH, and cortisol in hypoglycemia compensation

During fasting, extrahepatic tissue metabolism is fueled by glucose and ketone bodies, both produced in hepatocytes through glycogenolysis or gluconeogenesis and β-oxidation of fatty acids, respectively. During short-term fasting, hepatic glucose production proceeds through glycogenolysis, which is triggered by the decrease in insulin and fibroblast growing factor 19 and increase in glucagon. In long-term fasting, hepatic glucose production is performed through gluconeogenesis, triggered by the decrease in insulin and increase in glucagon, GH, and cortisol. Gluconeogenic substrates include α-ketoacids, such as pyruvate, α-ketobutyrate, α-ketovalerate, and α-ketoglutarate, which are derived from the respective α-amino acids. This conversion is mediated and promoted, among others, by the mitochondrial deacetylases Sirtuin3 and Sirtuin5, which increase during fasting. The gluconeogenic substrates also include glycerol, derived from triglycerides [15]. Triggered by falling insulin levels, some gluconeogenesis, out of glycerol, glutamine, and lactate, also occurs in the kidneys [16].

Pyruvate can either be converted to acetyl coenzyme-A by pyruvate dehydrogenase complex (PDC) and fed into the Krebs cycle, or channeled into gluconeogenesis. Pyruvate dehydrogenase kinases (PDKs, four isoforms 1–4) inactivate PDC through phosphorylation, directing pyruvate to gluconeogenesis.

Gluconeogenesis is dependent on the availability of the aforementioned substrates and the expression of the key gluconeogenic enzymes, phosphoenolpyruvate carboxykinase (PEPCK) and glucose-6-phosphatase (G6Pase). The expression of the key gluconeogenic and β-oxidation enzymes is triggered by the decrease in insulin and increase in glucagon, via several factors, including CREB (cAMP response element binding), CRTC2 (CREB-regulated transcription coactivator 2), FOX1 (Forkhead box protein O1), C/EBPα/β (CCAAT-enhancer-binding proteins α/β), PGC-1α (PPARα gamma coactivator-1alpha), and CBP–p300 (CREB binding protein p300). CCAAT (cytosine-cytosine-adenosine-adenosine-thymidine) is a box motif, present in several gene promoters, whereas PPARα (peroxisome proliferator activated receptor alpha) is the most important enhancer of fatty acid β-oxidation in mitochondria and peroxisomes [15].

Full transcriptional activation of PEPCK promoter requires the activation of glucocorticoid receptor (GR) by glucocorticoids (GC) and hepatic nuclear factor-4α (HNF-4α) by PGC-1a [17]. GR expression in hepatocytes is stimulated by the factor Yin Yang 1, which is elevated during fasting [15]. The GC/GR complex contributes to sufficient brain glucose supply by impairing skeletal muscle and adipose tissue glucose uptake [18]. In addition, SRC-1 (steroid receptor coactivator-1), through its interaction with GR/GC complex as a ligand, coactivates C/EBPα, promoting β-oxidation and expression of the key gluconeogenic genes [15].

The metabolic effects of GH are mediated through the pathway GH receptor/JK2/STAT5 (JK2, Janus kinase 2; STAT5, signal transducer and activator of transcription 5). GR/GC and CBP-p300 are coactivators of STAT5 transcriptional activity [18], thus augmenting the effect of GH. STAT5 promotes, among other pro-survival and anti-aging genes, the transcription of PEPCK and PDK4 genes, thus stimulating gluconeogenesis and channeling pyruvate from the Krebs cycle to gluconeogenesis [15].

These mechanisms emphasize the importance, cooperation, and interdependence of the actions of the four hormones *in glycogenolysis, gluconeogenesis, and β-oxidation, and the interconnections of gluconeogenesis and β-oxidation regulations*.

### 4.5 Insulin resistance and blood – cell interphase

For a deeper understanding of HOMA-IR decrease, the analysis of insulin's path from secretion to target cell might be useful. This path includes:

(A) Moving through the blood.
(B) Crossing the interphase between blood and target cell (blood – cell interphase), consisting of (B_1_) endothelial glycocalyx, (B_2_) endothelium, and (B_3_) interstitium.
(C) Interacting with the target cell [19].

To (A): Given the absence of any significant changes in blood pressure and heart rate during DF [1,7], the rate, at which insulin moves through the blood, was not expected to change.

To (B): Possible mechanisms, through which DF may affect the structure and function of the blood – cell interphase are: (β_1_) Improvement of structure and function of endothelial glycocalyx through (I) elevated cortisol, (II) increased oxygen supply, (III) reduced blood volume, and (IV) increased TAC (plasma antioxidant capacity) [20,21], all of which are given during DF [1]; (β_2_) Increase of the activity of endothelial nitrogen oxide synthase (eNOS), due to the increase in both cellular oxygenation and TAC [22], which are also given during DF [1]; and (β_3_) Increased insulin diffusion through the interstitium, due to the generalized edema elimination [1].

To (C): Since fasting was previously shown to increase insulin's binding to its receptor [23], this mechanism may contribute to the decrease of insulin resistance during DF.

Given that the principal structural and functional barrier in insulin's path from secretion to target cell is the endothelium [19], the mechanisms (β_1_) and (β_2_) may be crucial for the improvement in blood – cell interphase.

The blood – cell interphase improvement probably triggered the decrease in insulin resistance (Fig. 2B).

### 4.6 GH and IGF-1

Hypoglycemia has previously been shown to stimulate the secretion of GH via GHRH (growth hormone releasing hormone) [24]. The centrally directed GH pulsatility and amplitude may selectively reduce intima and media thickness, resulting in anti-atheromatous effect and improved blood vessel physiology [25]. Beyond its essential metabolic actions (see §4.4), GH, via IGF-1, acts as a tissue-protecting hormone [26].

GH and insulin stimulate the production of IGF-1 in the liver. Beyond its well-known anabolic action, IGF-1, through its antioxidant effects on mitochondrial metabolism, exerts pro-survival, anti-aging, and tissue-protective actions [26]. Among the tissues protected are the myocardium [27], bones, and skeletal muscles [28]. Therefore, we assume that the observed increase in IGF-1 contributed to the tissue protection shown here (Fig. 6).

Multiple studies have documented that IGF-1 levels decreases upon caloric restriction in mammals and this reduction is refractory to GH-increase [29,30]. This expected adaptation is attributed to the deacetylase Sirtuin1, which increases during fasting. Sirtuin1 deacetylates STAT5, thereby suppressing the GH-dependent IGF-1 production in the hepatocytes [31].

Consequently, a substantial decrease in IGF-1 was expected during DF. Instead, a significant increase was observed. The underlying mechanism of this atypical IGF-1 response remains unclear. We hypothesize that the increase in IGF-1 during DF played an essential role in protecting functional tissues from damage in an energy- and water-deprived state. However, the finding warrants confirmation and further investigation in future studies.

Formation and progression of malignancies have been linked, among other factors, to IGF-1, under the precondition of a concomitant increase in insulin [32]. In light of the observed massive decrease in insulin, this link does not apply in this context.

### 4.7 Hypothalamic-pituitary-adrenal (HPA) axis

The upregulation of HPA axis during DF has been previously studied [1] and discussed above (§4.4). It has to be noted, that, in addition to cortisol, vasopressin also has anti-hypoglycemic actions [1].

### 4.8 Cholesterols and triglycerides

#### 4.8.1 Cholesterols

The selective increase in LDL-C during DF, alongside the non-significant change of HDL-C, constitutes an important finding of this paper. Considerable cholesterol elevations were also reported following both prolonged fasting [33] and massive weight loss [34], while HDL stability has been observed during intermittent fasting and energy-restricted eating [35]. The observed LDL-C increase during DF raises the question of whether it resulted from de-novo synthesis or mobilization of reserves.

SREBPs (sterol regulatory element-binding proteins) are transcriptional factors, that regulate the transcription of LDLr (LDL-receptor) and proteins critical for the biosynthesis of both cholesterol and triglycerides. The SREBP-1 isoform-family preferentially modulates the de novo biosynthesis of fatty acids and triglycerides, while the SREBP-2 isoform-family that of LDLr and cholesterol [36]. The hormone sensitive isoform SREBP-1c regulates the biosynthesis of both triglycerides and cholesterol [15]. Fasting was previously shown to downregulate both isoform-families, and, through the decrease in insulin and increase in glucagon, SREBP-1c as well [15,36]. Accordingly, the biosynthesis of fatty acids, triglycerides, cholesterol, and LDLr is inhibited and the LDLr-mediated uptake of cholesterol by hepatocytes during DF is suppressed.

Hence, the substantial increase in LDL-C, observed here, can only be a result of mobilization from cholesterol ester depots.

Cholesterol esters are stored in lipid droplets found in various cells such as adipocytes, hepatocytes, and macrophage foam cells. Hydrolysis of cholesterol esters in lipid droplets of macrophage foam cells is enhanced through the decrease in insulin [37].

In the process of reverse cholesterol transport (RCT), free cholesterol and phospholipids efflux from peripheral cells (e.g., macrophage foam cells) initially via ABCA1 (ATP Binding Cassette A1) and subsequently via ABCG1/SRB1 (ATP Binding Cassette G1/Scavenger Receptor B1) and are transferred to HDL. Cholesterol from circulating HDL-C is then transferred to hepatocytes via SRB1. There, cholesterol is converted into bile acids, which are excreted into the intestine [38]. The conversion of cholesterol into bile acids is intensified during fasting [39]. Other pathways, normally transferring cholesterol to hepatocytes via LDLr, such as CETP (cholesterol ester transfer protein) – VLDL – LDL or ApoE-HDL [38], are during DF likely redirected to TICE (Trans-Intestinal Cholesterol Efflux).

TICE is a non-LDLr mediated process of cholesterol efflux from circulating LDL-C through the intestinal wall into the lumen [40]. TICE appears to employ the ABCB5/ABCB8 (ATP-Binding Cassette B5/B8) to transfer cholesterol from the apical end of enterocytes into the lumen [39]. It is a turbo mechanism for cholesterol excretion [41] and is known to further intensify during fasting. During it, TICE may feed the metabolism of intestinal microbes and, through the microbe-enterocyte interaction, supply the enterocytes with luminal fuel [42].

The intensified TICE during fasting elucidates both the LDL-C increase during DF and its contribution to hypoglycemia compensation.

The enhanced elimination of cholesterol during DF via both bile acids and TICE necessitates a highly intensified cholesterol efflux from peripheral cells, such as macrophage foam cells. However, this intensification does not result in an HDL-C increase nor does an HDL-C increase imply an intensified cholesterol efflux from peripheral cells [38]. Thus, it appears that no change in HDL-C is necessary during DF, and in fact, no such change occurs.

#### 4.8.2 Triglycerides

Since SREBP-1 is suppressed during fasting, the de novo biosynthesis of fatty acids and triglycerides is also suppressed. Thus, the increasing β-oxidation, evidenced by the intensified acetoacetate excretion, must be fed by fatty acids, liberated from triglyceride depots.

Triglycerides are stored in lipid droplets of adipocytes, hepatocytes, and macrophage foam cells. Their hydrolysis proceeds in three steps to liberate one fatty acid at each one, catalyzed sequentially by ATGL (adipose tissue triglyceride lipase), HSL (hormone sensitive lipase), and MGL (monoacylglycerol lipase). ATGL is upregulated by fasting, cortisol, PPARα, and decreased insulin, MGL by fasting and HSL [43], and HSL by cortisol, glucagon, GH, and decreased insulin [37].

As mentioned above, the increase in glucagon and decrease in insulin stimulate the expression of CREB, which promotes the expression of PGC-1α. This in turn activates PPARα, stimulating fatty acid liberation and β-oxidation [15]. The resulting molecules are ketone bodies, leading to ketonemia. This leads to ketonuria, which was observed here.

The late increase in triglycerides observed during DF is an unexpected finding. This may be an effect of intensive lipolysis and subsequent liberation of abundant fatty acids. The metabolic pathway is probably the following: While a majority of fatty acids follows the known pathway of β-oxidation in hepatocytes, a minority follows a route deviating from the above mentioned regulation: They feed a low-level de novo triglyceride synthesis in hepatocytes. The newly synthesized triglycerides are then packed into VLDL and transported via the blood to fuel the metabolism of energy-deprived muscle cells [44].

As outlined above, DF can reduce the cholesterol ester and triglyceride deposits in adipose tissue, hepatocytes, and macrophage foam cells. It may represent a promising addition to therapeutic strategies aimed at reducing adipose tissue mass, NAFLD (non-alcoholic fatty liver disease), and atheromatosis. To implement such strategies, new, long-term, metabolic, DF-based diagrams, such as ADDF, need to be developed and tested.

### 4.9 Urine acetoacetate

Its increase pattern (Fig. 3) demonstrates the massive increase in triglyceride lipolysis, fatty acid β-oxidation and ketonemia during DF. As previously mentioned, ketone bodies are alternative fuels to glucose. Therefore, their increase during DF contributes substantially to hypoglycemia compensation.

### 4.10 Hypothalamic-pituitary-thyroid (HPT) axis and leptin

The pituitary-thyroid axis is controlled by TRH, which is produced by the parvocellular neurons in PVN. The axonal terminals of TRH-producing neurons project to the median eminence. From there, TRH is transported via portal circulation to the anterior pituitary [45].

Fasting inhibits the production of TRH. Neurons in the hypothalamic ventromedial nucleus relay output to PVN, inhibiting TRH synthesis [45,46]. Furthermore, fasting stimulates the enzyme pyroglutamyl peptidase II in tanycytes of median eminence, which degrades TRH [47]. The increase in cortisol [1] contributes to the suppression of TRH synthesis as well [48,49]. Through the downregulation of basic metabolic rate, the decrease in thyroid hormones substantially contributes to hypoglycemia compensation.

TSH inhibition during DF occurred more rapidly compared to fluid-only fasting [50], suggesting that the metabolic adaptation, although similar, proceeds at a faster rate during DF.

The spontaneous suppression of thyroid function during DF might impact the development of new approaches for the treatment of constitutional overheating and hyperhidrosis and borderline hypothyroidism.

Leptin production is stimulated by insulin and cortisol [51]. The well-documented decrease in leptin during fasting [52] is attributed to insulin decrease [53]. However, a biphasic behavior of this hormone was observed here. This was a new finding of current study. Although the initial decrease has been probably prompted by decreased insulin, the trigger of its late increase is unclear. We could only assume, that the enormous increase in cortisol on Days 4 and 5^1^ has induced this totally unexpected change.

### 4.11 Tissue enzymes

The insignificant changes in CPK and SGOT (primarily striated muscle cell enzymes) and the minimal changes of SGPT (primarily hepatocellular enzyme), γGT (primarily cholangiocytic enzyme), and LDH (an all-tissue cell enzyme, composed of isoenzymes originating, among others, from striated muscles, lungs, kidneys, pancreas, liver, brain, intestinal epithelium, and erythrocytes) imply that DF induced no detectable cellular damages to any body tissues.

However, an increased protein catabolism is required to feed gluconeogenesis and was implied by the rise in creatinine discharge in 24-h urine. Thus, the question of identity and origin of the catabolized proteins is raised.

Autophagy, involving damaged or too old intracellular organelles, is known to increase during fasting [54]. On the other hand, the mechanism of endocytosis and catabolism of damaged or too old extracellular glycoproteins is also known [55] and our data suggest, it intensifies during DF.

### 4.12 Summary of current findings

Below, we summarize the findings of this paper on DF.

I. Euglycemia resulted from **(1)** the upregulation of (a) vasopressin and cortisol, (b) hypothalamic-sympathetic system efferent outflow, (c) noradrenaline, (d) dopamine [1], (e) glucagon, (f) GH, and (g) ketonemia; and **(2)** the downregulation of (a) insulin and (b) HPT-axis and metabolism.
II. Tissue protection, demonstrated in the minimal or insignificant changes of tissue enzymes, probably resulted from the increase in IGF-1.
III. Insulin resistance decrease (Fig. 2), likely resulted from the improvement in blood – cell interphase.
IV. Possible improvement in arterial physiology, indicated by: **(1)** the decrease in insulin and **(2)** the increase in (a) GH and (b) LDL-C.
V. Decrease in body weight and circumferences (Table 3), most probably resulted from the massive edema elimination [1].

### 4.13 Conclusion

This work represents the first comprehensive analysis of the endocrine and biochemical mechanisms underlying hypoglycemia compensation during DF. A key finding was the observed reduction in insulin resistance during DF. The results encourage the further exploration of DF's role in the treatment of metabolic syndrome, type-2-diabetes, non-alcoholic fatty liver disease, obesity, adiposity, and atheromatous disease.

### 4.14 Limitations of current study

The participants in current study were healthy and their number was small. Therefore the transfer of our conclusions on patients has to procced cautiously. DF must be serially applied on bigger groups of healthy volunteers, small groups of patients, e.g., obese ones or type-2-diabetics, bigger patient groups, and at the end in more populous groups and in various diseases. However, if a DF-experienced physician monitors, both clinically and through laboratory parameters, the fasting persons, there are no real risks for patients or healthy participants performing DF. Under these conditions, any reluctance or hesitation to apply DF should be cleared off.

### 4.15 Perspectives of DF

Even compensated, hypoglycemia, hypovolemia, and hypertonicity make medical supervision obligatory and limit the application time of this method to some days. Thus, the method per se can only have short-term effects. It is obvious, that the compensating mechanisms require intact endocrine and renal functions. Thus, individuals with pituitary, adrenal or renal insufficiency should not participate in multiple-day DF, whereas insulin- or thyroid hormone-dependent patients have to reduce their doses and be closely monitored.

Further research is required for.

- Planning DF-based schemes of prolonged duration, such as the ADDF, enabling long-term effects.
- Specifying and testing the appropriate metabolic schema for each particular disorder.
- Describing the complete therapeutic spectrum of DF, and
- Registering possible, not yet detected, side effects.

### 4.16 Notice about the availability of data

The measurements-data used to support the findings of this study are included within the supplementary files under the names “Data file1” and “Data file2”.

## CRediT authorship contribution statement

**Ioannis-Eleemon Papagiannopoulos-Vatopaidinos:** Writing – review & editing, Writing – original draft, Validation, Project administration, Methodology, Investigation, Data curation, Conceptualization. **Maria I. Papagiannopoulou:** Writing – review & editing, Writing – original draft, Validation, Investigation, Data curation, Conceptualization. **Eleni N. Dotsika:** Visualization, Supervision, Methodology, Investigation, Data curation, Conceptualization.

## Ethics

The collection of data for current study was performed in cooperation with the Hellenic Pasteur Institute in Athens. At the time of data collection (May 2014), no bioethical committee was active in the Institute. According to the Certification Ref. No. 1510 (February 17, 2022) of Director General of Hellenic Pasteur Institute, 157 Vas. Sofias Avenue, Athens 11521, Greece, the first decision for constituting an Institutional Review Board for Bioethics was made 2016.

## Funding

We received no grant from any funding agency in the public, commercial, or not-for-profit sector.

## Authors’ contribution statement

P-V, I-E contributed to conceptualization and acquisition, analysis, and interpretation of data, first draft, and critical revision of the paper; P, MI contributed to conceptualization and analysis and interpretation of data, first draft, and critical revision of the paper; D, EN contributed to conceptualization, analysis, interpretation of data, and first draft of the paper. All three authors approved finally the version submitted.

## Declaration of interests

We have no conflicts of interests to declare.
