# Supplementary material for: Hypoglycemia compensation mechanisms in dry fasting
Source: Metabol Open. 2025 Apr 15;26:100363. doi: 10.1016/j.metop.2025.100363 (PMC12206320; doi:10.1016/j.metop.2025.100363)
Supplement: Multimedia component 3 [file mmc3.pdf]

HELLENIC REPUBLIC
MINISTRY OF DEVELOPMENT AND INVESTMENTS
GENERAL SECRETARIAT FOR RESEARCH & INNOVATION (G.S.R.I.)

ΕΛΛΗΝΙΚΟ ΙΝΣΤΙΤΟΥΤΟ ΠΑΣΤΕΡ
INSTITUT PASTEUR HELLENIQUE

Athens, 17.02.2022
Ref.No. 1510

To whom it may concern

The Hellenic Pasteur Institute (H.P.I.), based in 127 Vass. Sofias Ave., Athens 11521, Greece, is a biomedical research institute characterized by scientific excellence. It is herein after certified that in 2014, the H.P.I. did not have any institutional Review Board for Bioethics in order to approve scientific collaborative protocols of the researchers of the Institute.

The Executive Board (ExBo) decision no 279/01.02.2016 and the Director's General decision no 1441/09.05.2016 constituted a seven-member "Bioethics Committee". In the ExBo decision no 4030/30.07.2018, the aforementioned Committee was harmonized to the requirements of the relevant Greek legislation, as defined by law 4521/2018, and consequently the previous "Bioethics Committee" was reconstituted and renamed as "Research Ethics Committee".

The researchers of the Hellenic Pasteur Institute, until 2016, had the academic freedom to participate in collaborative research studies/clinical trials related to their scientific interest and expertise, relying on the Declaration of the Principal Investigator of the study.

Digitally signed by EFSTATHIOS GKONOS
Date: 2022.02.17 12:29:34 EET

Dr Efstathios Gonos

Director General of the Hellenic Pasteur Institute

Λεωφ. Βασιλίσσης Σοφίας 127, Αθήνα 115 21 ● 210 64 78 800 ● Fax: 210 64 25 038 ● e-mail: secr.director@pasteur.gr ● www.pasteur.gr
127, Vas. Sofias Ave., 115 21 Athénes, Gréce ● Tel: +30 210 6478800 ● Fax: +30 210 6425038 ● email: secr.director@pasteur.gr ● www.pasteur.gr
