# Supplementary material for: Hypoglycemia compensation mechanisms in dry fasting
Source: Metabol Open. 2025 Apr 15;26:100363. doi: 10.1016/j.metop.2025.100363 (PMC12206320; doi:10.1016/j.metop.2025.100363)
Supplement: Multimedia component 4 [file mmc4.docx]

Ioannis-Eleemon Papagiannopoulos-Vatopaidinos, MD, GP

Vatopaidi Monastery Hospital, GR 63086

Email: giannis@fastingtherapy.gr

Vatopaidi Monastery Hospital 02.08.2024

**DECLARATION OF THE PRINCIPAL INVESTIGATOR**

I, the below signing Principal Investigator of the submitted for publication study entitled

“Hypoglycemia Compensation Mechanisms in Dry Fasting”,

declare that the followed protocol fulfilled all criteria of Good Practice as well as all the necessary requirements of bioethics and medical deontology.

I also declare that the blood and urine samples were collected from 10^th^ to 19^th^ of May 2014. As it is certified by the Hellenic Pasteur Institute (Ref. No. 1510) from 17.02.2022), to that time no Bioethical Committee was acting.

I further declare that all participants were informed regarding the following:

1. They were free to accept or reject the participation in the study.
2. The participation will result into no risk for their health and no financial cost.
3. Purpose of the measurements was the evaluation of the changes of biochemical and endocrine parameters during dry fasting.
4. Their blood and urine samples will be used exclusively for scientific and research purposes and their anonymity will always be held.
5. They will be daily clinically examined and their blood pressure, oxygen saturation, and heart rate will be recorded by myself as General Practitioner. All critical renal and biochemical parameters will be daily measured by a Biopathology Physician and the obtained results of their biological samples will be at their disposal.
6. Their results can be destroyed and not considered in the study, whenever they apply for it.

After they were informed, the participants signed a written consent prior to data collection.

Ioannis-Eleemon Papagiannopoulos-Vatopaidinos MD, GP

Vatopaidi Monastery Hospital

Email: giannis@fastingtherapy.gr
